# FAAH as a Molecular Regulator of Endocannabinoid Signaling: Mechanistic Insights into Reproductive, Metabolic, and Inflammatory Dysfunctions in Polycystic Ovary Syndrome

**DOI:** 10.3390/molecules31121996

**Published:** 2026-06-07

**Authors:** Qinghe Lin, Defan Wang, Pingting Guo, Zhenghong Zhang, Zhengchao Wang

**Affiliations:** 1 Provincial Key Laboratory for Developmental Biology and Neurosciences, Key Laboratory of Optoelectronic Science and Technology for Medicine of Ministry of Education, College of Life Sciences, Fujian Normal University, Fuzhou 350007, China; qinghelin2025@163.com (Q.L.); pingtingguo@fafu.edu.cn (P.G.); 2 Fujian Provincial Key Laboratory of Reproductive Health Research, School of Medicine, Xiamen University, Xiamen 361102, China; defanwang@126.com; 3 College of Animal Science, Fujian Agriculture and Forestry University, Fuzhou 350002, China

**Keywords:** fatty acid amide hydrolase, polycystic ovary syndrome, hormonal dysregulation, inflammatory response, metabolic homeostasis

## Abstract

Fatty acid amide hydrolase (FAAH) is a critical metabolic enzyme in the endocannabinoid system. Through regulating the metabolism of lipid signaling molecules, FAAH is involved in a variety of physiological and pathological processes, including cell proliferation, inflammatory responses, and metabolic homeostasis. Polycystic ovary syndrome (PCOS), one of the most common endocrine and metabolic disorders affecting women of reproductive age, is closely associated with abnormal follicular development, dysregulated hormone secretion, insulin resistance, dyslipidemia, and inflammatory imbalance. Emerging evidence suggests that FAAH-mediated dysregulation of endocannabinoid metabolism is associated with the pathogenesis of PCOS through the modulation of inflammatory pathways, insulin sensitivity, and hormone secretion. This review systematically summarizes the structure, biological functions, and regulatory mechanisms of FAAH, with particular emphasis on its roles in PCOS-associated pathological processes, including reproductive dysfunction, hormonal dysregulation, metabolic imbalance, and inflammatory responses. This review aims to provide a theoretical foundation for elucidating the molecular mechanisms underlying PCOS and for the development of novel therapeutic strategies.

## 1. Introduction

Fatty acid amide hydrolase (FAAH) is a key metabolic enzyme within the endocannabinoid system (ECS). It primarily regulates the biological activity of fatty acid amide signaling molecules through hydrolysis, such as AEA. Thereby, FAAH can maintain the dynamic equilibrium of endocannabinoid signaling [1,2]. The ECS is a critical lipid signaling regulatory system, principally composed of endocannabinoids (AEA and 2-AG), cannabinoid receptors (CB1 and CB2), and associated synthesis and degradation enzymes. And it plays pivotal roles in neuromodulation, immune responses, and the maintenance of metabolic homeostasis [1]. The ECS exhibits a characteristic “retrograde synaptic signaling” regulatory mechanism: postsynaptic neurons synthesize and release endocannabinoids, which subsequently act upon CB1 or CB2 receptors located on the presynaptic membrane, consequently modulating neurotransmitter release and neuronal excitability [1].

Within this system, FAAH serves as one of the core metabolic enzymes regulating ECS activity. Ueda et al. found that FAAH primarily catalyzes the hydrolysis of the endocannabinoid AEA into arachidonic acid and ethanolamine, which may terminate signal transmission and maintaining the dynamic equilibrium of ECS signaling levels [2]. Ren et al. found that FAAH, in conjunction with another major degradation enzyme MAGL participates in the metabolic regulation of endocannabinoids. Specifically, FAAH is primarily responsible for the degradation of AEA, while MAGL is primarily involved in the hydrolysis of 2-AG. Consequently, these two enzymes collectively maintain the stability of the ECS lipid signaling network [3,4]. Recent research has revealed that inhibiting FAAH can elevate endocannabinoid levels, which may indirectly enhance ECS signaling activity [3]. This approach has demonstrated potential therapeutic value across a range of pathological processes, including pain regulation, inflammatory responses, anxiety and depression, and neurodegenerative diseases [3,4]. Furthermore, as research in medicinal chemistry has advanced, a multitude of FAAH inhibitors with diverse structural scaffolds have been developed, including amide, carbamate, and urea derivatives. These compounds have exhibited significant analgesic and anti-inflammatory effects in animal models, resulting in forward research into FAAH as a potential drug target [5,6]. Therefore, FAAH serves as a key enzyme within the metabolic regulatory network of the ECS. It is also considered an important regulatory node that may connect endocannabinoid signaling with a wide range of physiological and pathological processes. Consequently, research into its function holds significant importance for elucidating the mechanisms by which the ECS operates in the context of neurological and metabolic diseases, as well as for developing novel therapeutic strategies.

Polycystic ovary syndrome (PCOS) is a multifactorial endocrine disorder affecting women of reproductive age. It is clinically characterized by hyperandrogenism, oligo- or anovulation, and polycystic ovarian morphology [7]. The pathophysiology of PCOS involves interactions among genetic predisposition, endocrine disruption, and metabolic abnormalities, such as insulin resistance and chronic low-grade inflammation, which collectively contribute to reproductive and metabolic dysfunction [8]. Emerging evidence suggests that ECS and its regulatory enzyme FAAH may play important roles in these processes.

During recent years, Witchel and colleagues. have proposed a pathological framework characterized by phenotypic heterogeneity and multi-system interactions; but controversies regarding diagnostic criteria, genetic mechanisms, and causal pathological relationships remain unresolved [9,10,11,12,13,14]. Using a maternal–fetal–offspring model, Witchel et al. demonstrated the pivotal role of the intrauterine environment in the intergenerational transmission of PCOS [9]. Environmental factors may exacerbate metabolic and hormonal imbalances, contributing to familial aggregation and an estimated heritability of approximately 70%. These factors include obesity, unhealthy lifestyle patterns, and exposure to endocrine-disrupting chemicals [9]. Rosenfield et al. proposed the concept of tissue-selective insulin resistance to explain the metabolic abnormalities associated with PCOS. Specifically, hyperinsulinemia stimulates androgen production while suppressing the synthesis of sex hormone-binding globulin (SHBG) [10].

Furthermore, chronic low-grade inflammation is recognized as an important contributor to PCOS pathogenesis. Elevated levels of inflammatory cytokines and macrophage infiltration into adipose tissue can reduce insulin sensitivity and impair follicular development [10]. Endocrine abnormalities in PCOS include the dysfunction of hypothalamic–pituitary–ovarian (HPO) axis, excessive adrenal androgen secretion, and elevated anti-Müllerian hormone (AMH) levels, all of which contribute to the arrest of follicular development [11]. Pasquali and colleagues further suggested that activation of the sympathetic nervous system may contribute to PCOS pathogenesis; for example, increased ovarian sympathetic nerve fiber density and overexpression of nerve growth factor (NGF) may promote androgen production [12]. In addition, dyslipidemia may further aggravate these metabolic and endocrine disturbances [13,14].

Despite substantial progress, several limitations remain in current PCOS research. For example, the diagnostic criteria for androgen assessment and ultrasonographic evaluation have not yet been fully standardized, no major causative genes have been conclusively identified, and data from studies involving specific patient populations and long-term intervention follow-up remain limited [15]. Therefore, future studies should prioritize the standardization of diagnostic criteria, strengthen multi-omics integration analyses, and establish large-scale cohorts for specific populations to further elucidate the mechanisms underlying PCOS pathogenesis and optimize the strategies of disease management [16].

Notably, FAAH expression is significantly decreased in the endometrial tissue of PCOS patients and exhibits phase-specific variation throughout the menstrual cycle [17]. Specifically, FAAH expression levels are significantly lower than those in healthy women during the proliferative and secretory phases, whereas no significant difference is observed during the menstrual phase. These findings suggest that FAAH expression is closely associated with cyclical reproductive physiological changes [17]. Reduced FAAH expression may result in the elevated levels of endocannabinoid anandamide (AEA). Because a physiological reduction in endocannabinoid levels is considered favorable for embryo implantation, aberrant FAAH expression may contribute to impaired post-ovulatory embryo implantation in PCOS patients [7]. Furthermore, the ECS is widely distributed throughout the HPO axis and female reproductive tissues. Through the activation of cannabinoid receptor 1 (CB1), the ECS participates in the regulation of insulin secretion, energy metabolism, and endocrine function, all of which are closely associated with the pathophysiology of PCOS [7]. Cui et al. have also demonstrated a positive correlation between FAAH expression and the severity of insulin resistance, suggesting that FAAH may participate in the regulation of metabolic dysfunction in PCOS [18].

In addition, alterations in ECS signaling can affect luteinizing hormone (LH) secretion patterns and follicular development, and are closely associated with the obesity and energy metabolism disorders frequently observed in PCOS patients [17]. Therefore, as a critical regulatory component of the ECS, FAAH appears to contribute to PCOS pathogenesis through modulation of endocannabinoid levels, consequently influencing metabolic homeostasis, inflammatory responses, and reproductive function. However, the precise molecular mechanisms and regulatory networks underlying these effects remain incompletely understood. Accordingly, this review summarizes the alterations of FAAH expression during PCOS progression and discusses its potential regulatory mechanisms, with the aim of providing a theoretical basis for understanding PCOS pathogenesis and identifying novel diagnostic and therapeutic targets.

## 2. FAAH and Endocannabinoid Signaling: Molecular Structure and Physiological Functions

**Figure 1:**
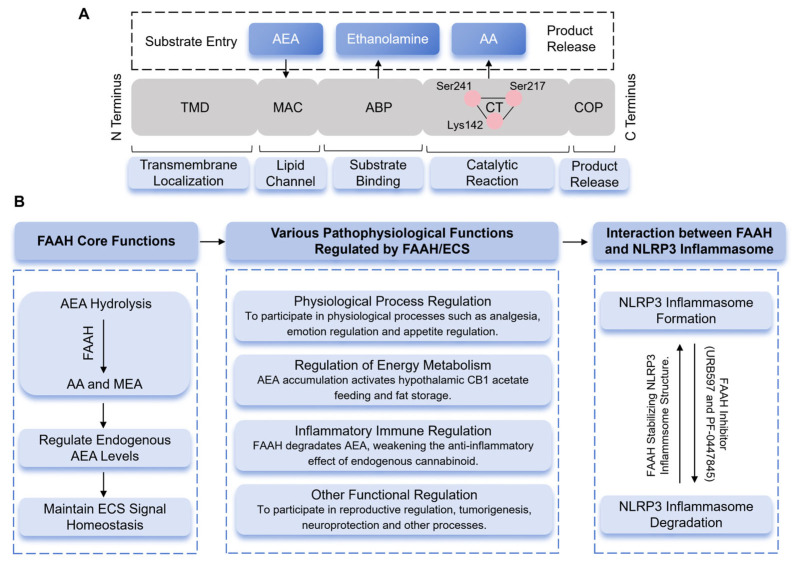
FAAH structure and its role in ECS regulation. (**A**) The molecular structure of FAAH. (**B**) The tole of FAAH in the regulation of ECS homeostasis.

FAAH belongs to the amidase signature (AS) superfamily of hydrolases, and its encoding gene is located on human chromosome 1p35–p34, making FAAH one of the most extensively studied members of this enzyme family [19]. FAAH exists as a homodimer, with each monomer anchored to the endoplasmic reticulum membrane through an N-terminal transmembrane domain. A lateral α-helix forms a membrane access channel that facilitates the efficient entry of lipid substrates into the catalytic site of the enzyme. The active site of FAAH consists of a membrane access channel (MAC), an acyl-chain binding pocket (ABP), and a cytosolic portal (Figure 1A). The catalytic activity of FAAH depends on a Ser241–Ser217–Lys142 catalytic triad that mediates substrate hydrolysis, whereas hydrogen bonding and hydrophobic interactions stabilize substrate binding and ensure catalytic efficiency [19].

Furthermore, several functionally distinct FAAH-related isoforms have been identified, including FAAH-1, FAAH-2, and the lysosome-localized N-acylethanolamine acid amidase (NAAA). FAAH-1 is predominantly expressed in the central nervous system, while FAAH-2 is mainly expressed in human adipose tissue. Collectively, these enzymes participate in the degradation and metabolic regulation of endocannabinoids and related lipid signaling molecules [20]. FAAH also exhibits functional genetic polymorphisms. In particular, the rs324420 polymorphism has been reported to reduce protein stability and enhance endocannabinoid signaling activity. This polymorphism has been associated with multiple phenotypic traits, including alcohol consumption behavior and sleep-related characteristics [21]. FAAH is widely expressed in metabolic and reproductive tissues, including the liver, adipose tissue, ovaries, and endometrium. Its expression is regulated by hormones, inflammatory cytokines, and transcription factors and may also be influenced by exogenous factors, such as chronic alcohol consumption and cannabis exposure [22].

FAAH is an essential enzyme responsible for the degradation of endocannabinoids and may play an important role in maintaining endocannabinoid homeostasis [20]. It hydrolyzes key ligand AEA into arachidonic acid (AA) and ethanolamine (MEA), thereby regulating signaling through CB1 and CB2 receptors (Figure 1B). Alterations in FAAH expression or activity have been associated with multiple physiological processes, including reproductive and metabolic regulation. including pain modulation, mood regulation, appetite control, and immune responses. For example, in migraine-associated neural regions, FAAH cooperates with MAG in endocannabinoid degradation, which may influence neurotransmission and neurodevelopment through modulation of AEA signaling [23]. FAAH also plays an important role in energy metabolism. Reduced FAAH activity is associated with increased AEA levels leads to AEA accumulation and subsequent activation of hypothalamic CB1, thereby promoting food intake and lipid storage [18,19]. In addition, FAAH influences adipocyte differentiation and lipid metabolism through modulation of endocannabinoid signaling in adipose tissue. For instance, in obesity and metabolic syndrome models, aberrant FAAH expression can impair insulin signaling pathways and exacerbate insulin resistance, whereas pharmacological inhibition of FAAH has been shown to improve insulin sensitivity and reduce hepatic lipid accumulation [24,25].

In the context of inflammation and immune regulation, endocannabinoids exert intrinsic anti-inflammatory effects, which can be attenuated by FAAH-mediated degradation of AEA [26]. Furthermore, FAAH is involved in reproductive regulation, tumorigenesis, and neuroprotection. For example, in the ovaries and endometrium, FAAH regulates follicular development, ovulation, and embryo implantation through modulation of AEA levels [19]. In PCOS patients, decreased FAAH expression may lead to excessive AEA accumulation and impaired embryo implantation [18,27,28]. In the models of cancer and neurodegenerative diseases, FAAH inhibition has been shown to restore the antitumor and neuroprotective effects of endocannabinoids [29]. Finally, FAAH has been reported to interact with and stabilize the NLRP3 inflammasome complex (Figure 1B). Conversely, FAAH inhibitors, such as URB597 and PF-04457845, can disrupt this interaction and promote NLRP3 degradation, resulting in suppressing inflammasome activation [30].

## 3. Pathophysiological Basis of PCOS Relevant to Endocannabinoid Signaling

The hallmark features of PCOS include chronic oligo-ovulation or anovulation, hyperandrogenism, and polycystic ovarian morphology, and the disorder is frequently accompanied by insulin resistance and metabolic dysfunction. The prevalence of PCOS varies among different populations. Among Han Chinese women of reproductive age (19~45 years), the prevalence is approximately 5.6%, whereas the global prevalence ranges from 5% to 15%, depending on diagnostic criteria, ethnicity, and obesity prevalence. In women with obesity (BMI > 30 kg/m^2^), the prevalence may increase to approximately 14% [9,31]. PCOS accounts for approximately 75% of cases of anovulatory infertility, and is considered the leading cause of infertility associated with ovulatory dysfunction. Among adolescents, the prevalence is approximately 6%, with menstrual irregularities and hyperandrogenism typically representing the initial clinical manifestations [9,31]. The primary clinical manifestations of PCOS include menstrual irregularities, hyperandrogenemia, polycystic ovarian morphology, and infertility associated with ovulatory dysfunction [32]. Furthermore, PCOS patients frequently exhibit clinical signs of insulin resistance, such as central obesity and acanthosis nigricans, and are at an increased risk of psychological disorders, including depression and anxiety [33]. In the long term, PCOS significantly increases the risk of metabolic syndrome, type 2 diabetes mellitus, hypertension, endometrial cancer, non-alcoholic fatty liver disease, and obstructive sleep apnea. Notably, the risk of metabolic syndrome is increased by approximately twofold, whereas the risk of endometrial cancer may increase by nearly fourfold [34,35].

**Figure 2:**
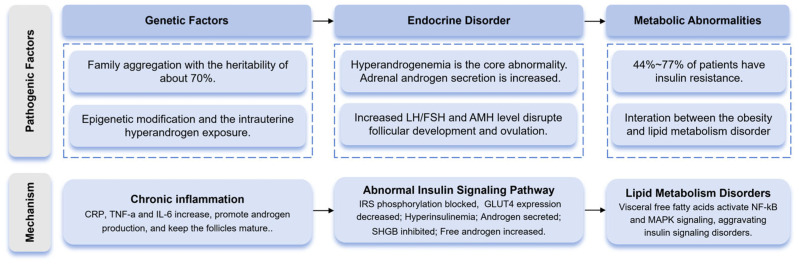
The pathogenic mechanism of multi factor synergy in PCOS. This schematic diagram shows the pathogenic factors and the molecular mechanisms of PCOS.

The pathogenesis of PCOS is characterized by complex multifactorial interactions involving a multilayered regulatory network that encompasses genetic susceptibility, endocrine dysfunction, and metabolic abnormalities (Figure 2). Ajmal et al. have demonstrated that PCOS exhibits significant familial aggregation and is closely associated with polymorphisms in genes involved in insulin signaling and steroid hormone synthesis. GWAS have identified multiple susceptibility loci, including THADA, FSHR, and DENND1A, with the estimated heritability of PCOS reaching approximately 70% [36]. Furthermore, epigenetic modifications and in utero exposure to hyperandrogenism may further increase disease susceptibility through transgenerational inheritance mechanisms [36]. From an endocrine perspective, hyperandrogenism represents a central feature of PCOS and is frequently accompanied by an elevated LH/FSH ratio, excessive adrenal androgen secretion, and increased AMH levels. Collectively, these alterations disrupt follicular development and ovulation [37]. In addition, approximately 44–77% of PCOS patients exhibit insulin resistance, which interacts synergistically with obesity and dyslipidemia to perpetuate a vicious cycle of metabolic dysfunction [32]. At the molecular level, the principal pathological mechanisms underlying PCOS involve chronic inflammation, insulin signaling dysregulation, and lipid metabolic disturbances. Elevated levels of inflammatory mediators, including CRP, TNF-α and IL-6, can promote androgen production while impairing follicular maturation [13]. Impaired phosphorylation of insulin receptor substrates reduces GLUT4 expression, which may contribute to insulin resistance. Moreover, compensatory hyperinsulinemia further stimulates ovarian and adrenal androgen secretion through the activation of PI-3K/Akt signaling pathway while suppressing SHBG synthesis, ultimately increasing circulating free androgen levels [15,38]. In addition, free fatty acids released from visceral adipose tissue can activate inflammatory signaling pathways, including NF-κB and MAPK, which may exacerbate insulin signaling dysfunction [13].

Collectively, PCOS is increasingly recognized as a systems-level disorder arising from dynamic interactions among genetic susceptibility, endocrine dysregulation, metabolic dysfunction, and chronic inflammation [39]. Rather than acting independently, these pathological processes form interconnected regulatory networks involving reproductive, metabolic, and immune pathways. Emerging evidence suggests that FAAH/ECS signaling may function as an important regulatory node within these networks, potentially linking ovarian dysfunction, insulin resistance, inflammatory activation, and endocrine imbalance. This systems-oriented perspective provides a conceptual framework for understanding the potential contribution of FAAH dysregulation to PCOS pathophysiology. 

## 4. Altered FAAH Expression and Endocannabinoid Dysregulation in PCOS

FAAH expression in ovarian tissue exhibits distinct cell-specific and spatial distribution patterns, and undergoes dynamic changes during different stages of follicular development [19]. Its localization and expression levels directly influence ovarian physiological functions, including follicular maturation and steroidogenesis. FAAH expression has been detected in oocytes within primordial, preantral, and antral follicles. But its expression level in oocytes is relatively low, suggesting that the surrounding AEA microenvironment is primarily regulated by adjacent somatic cells. FAAH is highly expressed in granulosa cells of dominant follicles, whereas its expression is markedly reduced in granulosa cells of atretic follicles. FAAH is also expressed in theca cells, although at lower levels than in granulosa cells, and these cell types contribute to the regulation of follicular development and local androgen production, respectively [19]. Furthermore, FAAH co-localizes with CB_2_ in granulosa cells, suggesting that it may indirectly regulate downstream signaling through AEA degradation [19]. In rat ovaries, FAAH co-localizes with CB_2_ receptors in oocytes and luteal cells and is also expressed in the ovarian surface epithelium [40]. In mouse ovaries, FAAH is widely expressed throughout ovarian tissue, and its expression can be regulated by the activation or inhibition of CB_1_/CB_2_. FAAH also participates in the degradation of endocannabinoids [41]. In human ovaries, FAAH is predominantly expressed in granulosa and luteal cells, which may contribute to the maintenance of local endocannabinoid homeostasis. Overall, the spatially restricted expression of FAAH within ovarian tissues suggests an important role in maintaining local endocannabinoid homeostasis. Current evidence indicates that FAAH may participate in the regulation of follicular development, steroidogenesis, and luteal function [41]. However, the precise cell-specific mechanisms remain incompletely understood and require further investigation.

Emerging evidence suggests that altered FAAH expression and associated ECS dysregulation may contribute to biological processes relevant to PCOS pathophysiology. However, whether FAAH dysregulation represents a primary pathogenic event or a secondary consequence of endocrine and metabolic abnormalities remains to be fully elucidated. Under physiological conditions, FAAH is widely expressed in secondary and tertiary follicles, corpora lutea, and corpora albicantia of the human ovary. By degrading AEA, FAAH participates in the regulation of follicular maturation and ovulation [19]. But in PCOS patients, FAAH expression is significantly reduced, leading to both local and systemic ECS dysfunction [42]. Histological studies have further confirmed that FAAH expression is significantly reduced in reproductive tissues of PCOS patients, particularly in the endometrium, compared with healthy controls [18]. This downregulation of FAAH leads to elevated AEA levels, which impair ovarian ovulation and luteal function, and are closely associated with metabolic and reproductive abnormalities in PCOS patients, including insulin resistance, obesity, and reduced clinical pregnancy rates [18]. Notably, treatment with a combination of Diane-35 and metformin can significantly restore the reduced expression of FAAH in reproductive tissues of PCOS patients [43]. This suggests that FAAH may serve as a promising biological target for improving the endocrine–metabolic profile of PCOS and for monitoring therapeutic efficacy [44].

Taken together, current evidence suggests that altered FAAH expression and ECS dysregulation may represent important features of the ovarian microenvironment in PCOS. However, the majority of available data derive from experimental models or associative clinical studies, and further investigations are required to clarify the temporal and causal relationships between FAAH dysregulation and disease progression.

## 5. Mechanistic Role of FAAH Dysregulation in PCOS Pathogenesis

FAAH is a major enzymatic regulator of endocannabinoid turnover and has attracted increasing attention in PCOS research. Current evidence suggests that alterations in FAAH expression or activity may influence several biological processes associated with PCOS, including follicular development, endocrine regulation, metabolic homeostasis, and inflammatory responses. However, most mechanistic evidence derives from experimental models or associative clinical observations, and direct causal relationships remain to be fully established.

### 5.1. The Effect of FAAH on Follicular Development and Ovulation

**Figure 3:**
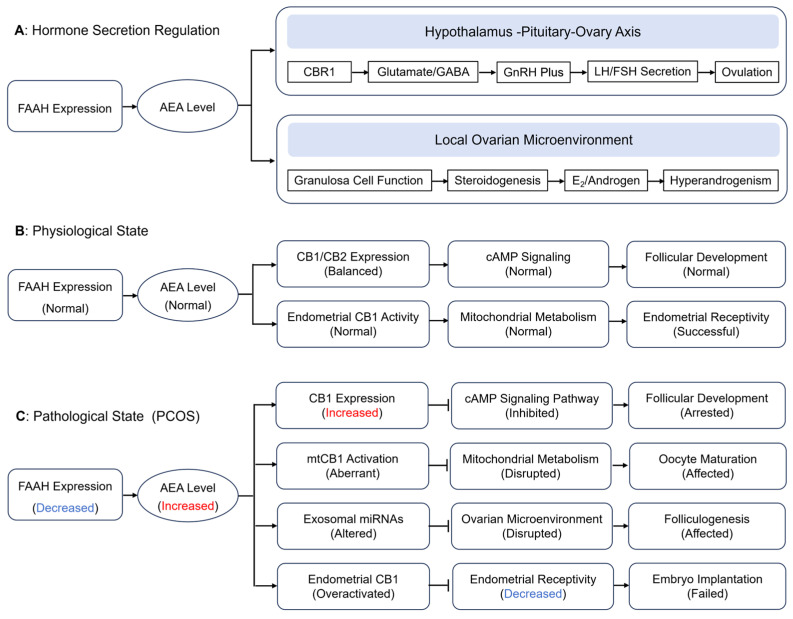
The contribution of FAAH/AEA signaling to hormonal imbalance, follicular development, metabolic alterations and inflammatory responses during the pathogenesis of PCOS. (**A**) The effect of FAAH/AEA on hormone secretion through HPO axis and local ovarian microenvironment. (**B**) The effect of FAAH/AEA on follicular development and endometrial receptivity under physiological conditions. (**C**) The effect of FAAH/AEA on reproductive functions under PCOS pathological conditions.

In the ovary and endometrium, FAAH precisely regulates follicular development and reproductive function via ECS signaling (Figure 3A–C). Key components of the ECS include CB1, CB2, FAAH and NAPE-PLD, which are widely expressed in human ovarian follicles, granulosa cells, and stromal tissues.

As a key endocannabinoid, AEA shows a positive correlation between its concentration in follicular fluid and oocyte maturity, and physiological levels of AEA may promote follicular development (Figure 3B). But in the ovaries of PCOS patients, FAAH expression is significantly reduced, resulting in the excessive accumulation of AEA. Elevated AEA levels have been associated with impaired ovulation-related signaling, altered dominant follicle selection, and follicular developmental arrest in experimental studies [45,46]. Nevertheless, the extent to which these observations directly contribute to human PCOS remains to be clarified. Concurrently, in granulosa cells of PCOS patients, CB1 expression is abnormally elevated, whereas CB2 expression remains relatively stable. This receptor imbalance further exacerbates the abnormalities of follicular development [19,47].

The ECS also influences oocyte quality through the regulation of mitochondrial function (Figure 3C). Under PCOS conditions, accumulated AEA aberrantly activates mitochondrial CB1 (mtCB1), disrupting ovarian cellular energy metabolism and exacerbating impaired oocyte maturation [43]. In the endometrium, FAAH expression fluctuates in a cycle-dependent manner, peaking during the secretory phase and reaching its lowest level during the menstrual phase. In PCOS patients, FAAH expression is significantly reduced during both proliferative and secretory phases, whereas endometrial CB1 activity is abnormally increased. This is associated with reduced endometrial receptivity [17,18,19].

Furthermore, FAAH dysregulation and AEA accumulation under PCOS state, together with the dysregulation of HIF-1α pathway, collectively trigger lysosomal dysfunction and collapse of the autophagy-lysosome axis. This cascade subsequently leads to impaired follicular development, ovulatory dysfunction, and hormonal secretion disorders, consequently acting as a critical nexus linking ECS imbalance to ovarian functional impairment in PCOS [48,49,50,51,52].

Additionally, miRNAs within follicular fluid-derived exosomes may be indirectly altered by FAAH and ECS dysregulation, resulting in anovulatory infertility and implantation failure in PCOS patients [53,54].

### 5.2. The Effect of FAAH on the Regulation of Hormone Secretion

Within HPO axis and local gonadal hormone secretion, FAAH may play an important role through ECS modulation. The ECS is critically involved in reproductive endocrine regulation, with its core mechanism involving CB1 activation in the GnRH neurons and the pituitary gland (Figure 3A). This activation reduces the release of GnRH-stimulating neurotransmitters, such as glutamate, while enhancing inhibitory GABA signaling, ultimately leading to decreased secretion of pituitary FSH and LH [19].

In PCOS patients, excessive ECS activation driven by reduced FAAH expression exacerbates HPO axis dysfunction, leading to an elevated LH/FSH ratio and ovulatory dysfunction (Figure 3A). Kabakchieva et al. have demonstrated that CB1 agonists directly suppress GnRH secretion, whereas CB1 knockout mice exhibit ovulatory abnormalities [55]. Within ovarian tissue, reduced FAAH expression is generally associated with increased AEA accumulation. Elevated AEA levels have been linked to impaired steroidogenesis in granulosa cells and may contribute to disturbances in the balance between estrogen and androgen production [45,46,47].

Notably, ECS alterations vary among different PCOS phenotypes. For instance, endocannabinoid levels in patients with phenotype A (hyperandrogenism, ovulatory dysfunction and polycystic ovarian morphology) are lower than those in patients with phenotype B (hyperandrogenism and ovulatory dysfunction and no polycystic ovarian morphology) [26,55]. Furthermore, the hyperandrogenic state in PCOS patients may establish a negative feedback loop by downregulating FAAH expression, consequently exacerbating endocannabinoid imbalance and perpetuating a self-sustaining pathological cycle [18].

### 5.3. Neuroendocrine Regulation of FAAH and ECS Signaling

Although the present review primarily focuses on peripheral FAAH regulation, increasing evidence highlights the importance of central neuroendocrine mechanisms in PCOS pathophysiology [56].

The hypothalamus serves as a critical integrator of reproductive and metabolic signals. ECS components, including CB1 receptors and FAAH, are widely expressed within hypothalamic nuclei involved in appetite regulation, energy homeostasis, and reproductive control [57]. Activation of hypothalamic CB1 signaling can modulate GnRH neuronal activity, alter gonadotropin secretion, and influence feeding behavior [57].

In PCOS, obesity, insulin resistance, and chronic inflammation may disrupt hypothalamic signaling pathways, which may contribute to neuroendocrine dysfunction [58]. Conversely, altered ECS activity may influence both reproductive and metabolic phenotypes through central regulatory mechanisms [58]. These bidirectional interactions suggest that FAAH-mediated ECS signaling may participate in the integration of peripheral metabolic signals with central reproductive control.

Future studies investigating tissue-specific FAAH regulation within the hypothalamus and other neuroendocrine regions may provide important insights into disease heterogeneity and treatment responsiveness in PCOS [59].

### 5.4. Brain-Derived Neurotrophic Factor as a Potential Integrative Mediator

Brain-derived neurotrophic factor (BDNF) has emerged as an important regulator linking energy metabolism, inflammation, and neuroendocrine function [60]. Beyond its classical role in neuronal survival and synaptic plasticity, BDNF participates in appetite regulation, glucose homeostasis, and reproductive endocrine control [60].

Recent studies in nutritional immunology have suggested that BDNF may function as a molecular bridge connecting metabolic status with inflammatory and endocrine pathways [61]. Altered circulating BDNF levels have been reported in several metabolic disorders characterized by insulin resistance and chronic inflammation [62]. Emerging evidence also suggests a potential association between BDNF signaling and reproductive dysfunction in women with PCOS [61].

Although direct evidence linking FAAH and BDNF remains limited, both pathways converge on mechanisms regulating energy balance, neuroinflammation, and hypothalamic function [63]. Therefore, BDNF may represent a promising candidate mediator within the broader FAAH–ECS–neuroendocrine regulatory network. Further mechanistic studies are required to clarify these interactions and evaluate their therapeutic implications.

### 5.5. The Effect of FAAH on Insulin Resistance and Metabolic Disorders

**Figure 4:**
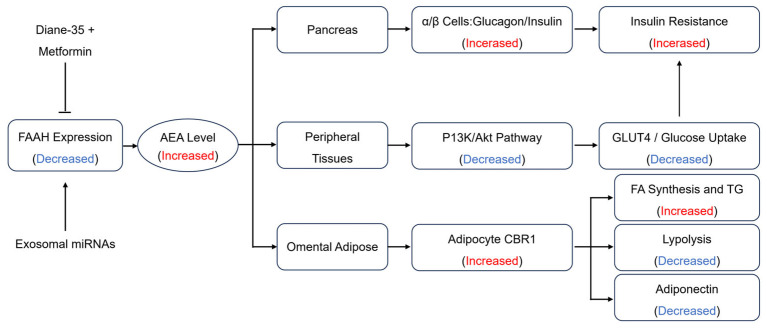
The effect of FAAH/AEA on insulin resistance and metabolic disorders in PCOS patients treated with or without Diane-35 and metformin.

FAAH has emerged as an important regulator of metabolic pathways associated with insulin resistance and lipid metabolism in PCOS (Figure 4). Accumulating evidence suggests that ECS hyperactivation may contribute to the development of insulin resistance in affected individuals [64]. Reduced FAAH activity is associated with increased AEA levels.

Elevated AEA concentrations may influence pancreatic endocrine function by stimulating β-cell insulin secretion and enhancing CB1-mediated signaling in α-cells, which may increase glucagon release [64]. Concurrently, accumulated AEA and 2-AG exacerbate insulin resistance through a dual mechanism. By interfering with PI-3K/Akt signaling downstream of insulin receptors in peripheral tissues such as the liver, skeletal muscle and adipose tissue, they suppress GLUT4 expression and reduce glucose uptake [19].

Adipose tissue is a major target organ of ECS, and CB1 expression is significantly upregulated in omental adipose tissue of PCOS patients (Figure 4). Through paracrine signaling, accumulated AEA activates CB1 receptors on adipocytes, promoting fatty acid synthesis and triglyceride accumulation, while inhibiting lipolysis. Furthermore, this process downregulates adiponectin secretion [19,65]. In obese PCOS patients, AEA levels are positively correlated with waist-to-hip ratio. Moreover, the obesity further increases adipose tissue levels of AEA and 2-AG [66], enhancing lipolysis and promoting free fatty acid accumulation [26,46,65,67].

Furthermore, FAAH may indirectly regulate metabolism-related miRNAs within exosomes, including miR-20b-5p and miR-143 [53,68]. Clinical data have demonstrated that combination therapy with Diane-35 and metformin can upregulate FAAH expression, reduce plasma AEA levels, and partially reverse CB1 overactivation, improving insulin sensitivity and lipid metabolism [17,26,46,69].

### 5.6. Lifestyle Factors, Energy Balance and FAAH Regulation

Lifestyle factors play a central role in the development, progression, and management of PCOS [70]. Dietary composition, obesity, physical activity, sleep quality, and energy balance profoundly influence insulin sensitivity, inflammatory signaling, and reproductive endocrine function [71]. Emerging evidence indicates that ECS activity is highly responsive to nutritional and metabolic status [72].

Obesity is associated with elevated circulating concentrations of AEA and 2-AG, enhanced CB1 signaling, and altered FAAH expression. Excessive ECS activation may promote appetite stimulation, adipogenesis, lipid accumulation, and impaired insulin signaling [19]. Conversely, weight reduction and lifestyle interventions have been shown to improve insulin sensitivity, attenuate inflammatory responses, and partially restore reproductive hormonal balance in women with PCOS [15,73].

Dietary composition may further influence ECS activity through alterations in fatty acid availability and downstream metabolic signaling pathways [73,74,75,76]. Similarly, regular physical activity may contribute to ECS homeostasis by improving mitochondrial function and reducing systemic inflammation [73,74,75,76,77,78,79].

Collectively, these observations suggest that FAAH dysregulation should be interpreted within a broader framework of metabolic adaptation and environmental influences. Lifestyle interventions may therefore complement pharmacological strategies targeting FAAH/ECS signaling.

### 5.7. The Effect of FAAH on Chronic Inflammatory Responses

**Figure 5:**
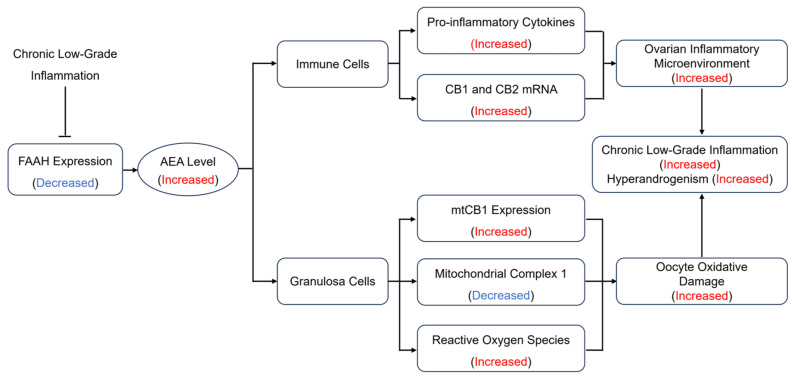
The effect of FAAH/AEA on chronic inflammatory responses through immune cells and granulosa cells under PCOS conditions.

Emerging evidence suggests that FAAH may participate in the complex interactions among hyperandrogenism, chronic low-grade inflammation, oxidative stress, and metabolic dysfunction observed in PCOS (Figure 5). However, the causal contribution of FAAH to these pathological interactions remains incompletely understood. The ECS has been implicated in inflammatory processes associated with PCOS through its effects on immune cell function [80]. Under physiological conditions, macrophages and lymphocytes express substantial levels of CB2 receptors. Activation of CB2 signaling by AEA has been reported to suppress the production of pro-inflammatory cytokines, including TNF-α and IL-6, thereby contributing to immune homeostasis [80].

In PCOS, chronic low-grade inflammation, indicated by elevated TNF-α and IL-6 and activation of NF-κB signaling, is associated with decreased FAAH expression. Concurrently, these inflammatory changes have been linked to enhanced AEA accumulation [19,68,80]. In this state (Figure 5), excessive ECS activation disrupts immune homeostasis. Notably, mRNA expression levels of CB1 and CB2 in peripheral blood monocytes are significantly increased, exacerbating a self-perpetuating cycle between inflammatory cytokines and endocannabinoids. These alterations may contribute to changes in the ovarian inflammatory microenvironment, and may adversely affect follicular development [19,68,80].

Furthermore, ECS dysfunction has been associated with mitochondrial dysfunction and enhanced oxidative stress in experimental models relevant to PCOS (Figure 5). In ovarian granulosa cells, reduced FAAH expression may increase AEA availability, which has been associated with enhanced mtCB1 signaling, impaired mitochondrial complex I activity, decreased adenosine triphosphate (ATP) production, and elevated reactive oxygen species (ROS) generation, amplifying oxidative stress-induced damage [45]. Shivyari et al. have demonstrated that in dehydroepiandrosterone-induced hyperandrogenic mouse models of PCOS, ovarian SOD activity is reduced, whereas ROS levels are elevated. But FAAH activation improves ECS function and increases GSH levels, mitigating oxidative stress-induced oocyte damage [81]. This self-perpetuating cycle is closely intertwined with hyperandrogenemia, and together they drive PCOS progression.

### 5.8. Systems-Level Integration of FAAH Dysregulation in PCOS

**Figure 6:**
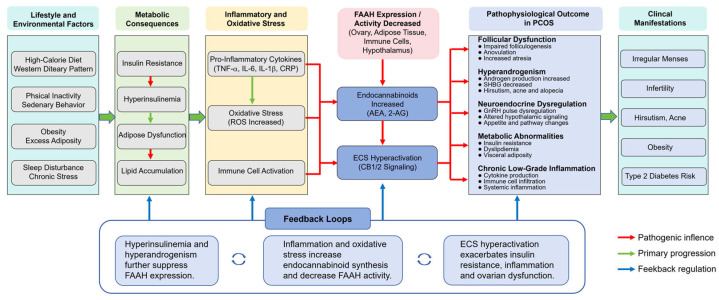
Systems-level model of FAAH dysregulation in PCOS.

PCOS is increasingly recognized as a complex systems disorder rather than a disease driven by a single pathogenic pathway [15]. Although endocrine dysfunction, insulin resistance, obesity, chronic inflammation, oxidative stress, and ovarian abnormalities are often discussed separately, growing evidence indicates that these processes form an interconnected regulatory network (Figure 6). Within this network, FAAH and the endocannabinoid system may function as an important integrative node linking metabolic, endocrine, inflammatory, and reproductive pathways [82].

Based on current evidence, a systems-level framework may be proposed in which reduced FAAH expression contributes to elevated endocannabinoid tone, particularly increased AEA accumulation [19]. Enhanced ECS activation may influence multiple biological processes simultaneously, including ovarian steroidogenesis, insulin signaling, adipose tissue metabolism, immune cell activation, and neuroendocrine regulation [83]. These effects are unlikely to occur independently; rather, they may reinforce one another through a series of positive feedback loops [83].

For example, insulin resistance promotes hyperinsulinemia, which stimulates ovarian androgen production. Hyperandrogenism may interact with FAAH/ECS signaling, potentially contributing to increased endocannabinoid tone and further ECS activation [19]. Similarly, obesity-associated adipose tissue dysfunction increases inflammatory cytokine production and endocannabinoid synthesis, whereas chronic inflammation may further reduce FAAH activity [19,84]. These interconnected loops may contribute to disease persistence and progression.

Therefore, FAAH dysregulation should not be viewed solely as a local ovarian abnormality but rather as a component of a broader regulatory network integrating reproductive, metabolic, and immune functions. Future investigations utilizing systems biology, multi-omics analysis, and network medicine approaches may provide a more comprehensive understanding of FAAH-mediated regulatory mechanisms in PCOS.

## 6. Conclusions

PCOS is increasingly recognized as a multifactorial and heterogeneous disorder arising from complex interactions among endocrine, metabolic, inflammatory, neuroendocrine, and environmental factors. Within this context, ECS has emerged as an important regulatory network involved in the coordination of reproductive function, energy homeostasis, immune responses, and metabolic adaptation. As the principal enzyme responsible for endocannabinoid degradation, FAAH plays a central role in maintaining ECS homeostasis and regulating local and systemic endocannabinoid signaling.

Accumulating evidence suggests that altered FAAH expression or activity may be associated with several biological processes relevant to PCOS pathophysiology, including follicular development, steroid hormone production, insulin sensitivity, lipid metabolism, oxidative stress, and chronic low-grade inflammation. However, the majority of currently available evidence derives from experimental models, observational studies, or indirect mechanistic investigations. Therefore, although FAAH dysregulation represents a promising area of research, its precise causal contribution to PCOS initiation and progression remains incompletely understood.

Importantly, FAAH should not be viewed solely as an ovarian regulatory factor. Rather, emerging evidence supports a broader systems-level perspective in which FAAH and ECS signaling may function as an integrative regulatory interface connecting metabolic dysfunction, endocrine imbalance, inflammatory activation, neuroendocrine regulation, and reproductive abnormalities. Through interactions with insulin signaling, adipose tissue metabolism, inflammatory pathways, hypothalamic regulation, and ovarian function, FAAH-mediated ECS activity may participate in multiple interconnected feedback networks that contribute to disease persistence and progression.

Lifestyle and environmental factors further add complexity to this regulatory framework. Obesity, dietary composition, physical inactivity, and altered energy balance are well-established contributors to PCOS and may influence ECS activity and FAAH expression. Consequently, FAAH dysregulation should be interpreted within the broader context of metabolic adaptation and environmental influences rather than as an isolated molecular abnormality. Future studies integrating lifestyle interventions with molecular investigations may provide valuable insights into the dynamic interactions between ECS signaling and PCOS progression.

From a translational perspective, FAAH represents a potentially attractive therapeutic target [85]. Nevertheless, current evidence remains largely preclinical, and significant challenges must be addressed before FAAH-targeted interventions can be translated into clinical practice. Future therapeutic strategies may benefit from combining modulation of FAAH/ECS signaling with established metabolic and lifestyle-based interventions, including weight management, dietary modification, physical activity, and insulin-sensitizing therapies. Such integrated approaches may offer greater efficacy than targeting individual pathways alone.

Overall, FAAH occupies a potentially important position at the intersection of reproductive, metabolic, inflammatory, and neuroendocrine regulation. Continued advances in multi-omics technologies, systems biology approaches, and network medicine are expected to provide deeper insights into FAAH-centered regulatory mechanisms and their relevance to PCOS. A more comprehensive understanding of these interconnected pathways may ultimately facilitate the development of more precise diagnostic tools, personalized therapeutic strategies, and improved clinical outcomes for women with PCOS.

## Data Availability

No new data were created or analyzed in this study. Data sharing is not applicable.

## Abbreviations

The following abbreviations are used in this manuscript:

2-AG	2-Arachidonoylglycerol
AA	Arachidonic Acid, ARA
ABP	Acyl Chain Binding-Pocket
AEA	Arachidonoyl Ethanolamide
AMH	Anti-Müllerian Hormone
Akt	Protein Kinase B, PKB
AS	Amidase Signature
ATP	Adenosine Triphosphate
BDNF	Brain-Derived Neurotrophic Factor
BMI	Body Mass Index
CB1	Cannabinoid Receptor 1, CNR1, CB1R
CB2	Cannabinoid Receptor 2, CNR2, CB2R
CBR1	Carbonyl Reductase 1
CRP	C-Reactive Protein
ECS	Endocannabinoid System
FAAH	Fatty Acid Amide Hydrolase
FoxO1	Forkhead Box O1
FSH	Follicle-Stimulating Hormone
GABA	Gama-Aminobutyric Acid
GLUT-4	Glucose Transporter-4
GnRH	Gonadotropin-Releasing Hormone
GSH	Glutathione
GWAS	Genome-Wide Association Study
HPOA	Hypothalamic-Pituitary-Ovarian Axis
IL-6	Interleukin-6
IRS	Insulin Receptor Substrate
LH	Luteinizing Hormone
MAC	Membrane Access Channel
MAGL	Monoacylglycerol Lipase
MAPK	Mitogen-Activated Protein Kinase
MEA	Monoethanolamine, Ethanolamine
miRNA	Micro Ribonucleic Acid, microRNA
NAAA	N-Acylethanolamine Acid Amidase
NAPE-PLD	N-Acylphosphatidylethanolamine-Hydrolyzing Phospholipase D
NF-κB	Nuclear Factor-κB
NGF	Nerve Growth Factor
PCOS	Polycystic Ovary Syndrome
PI-3K	Phosphoinositide-3 Kinase
PPAR	Peroxisome Proliferator-Activated Receptor
ROS	Reactive Oxygen Species
SHBG	Sex Hormone-Binding Globulin
SOD	Superoxide Dismutase
TNF-α	Tumor Necrosis Factor-Alpha
